# Pain management training for people with persistent pain and their informal carers (JOINT SUPPORT): multicentre randomised controlled feasibility trial with embedded qualitative study in English musculoskeletal services

**DOI:** 10.1136/bmjopen-2024-095069

**Published:** 2025-04-15

**Authors:** Toby Smith, Reema Khoury, Allie Welsh, Coralie Crowther, Sarah Hanson, Kelly Grant, Allan B Clark, Polly-Anna Ashford, Matthew Hammond, Martin Pond, Emma Dures, Jo Adams

**Affiliations:** 1 University of Warwick, Coventry, UK; 2 School of Health Sciences, University of East Anglia, Norwich, UK; 3 Norwich Medical School, University of East Anglia, Norwich, UK; 4 Cambridge University Hospitals NHS Foundation Trust, Cambridge, UK; 5 Leeds Beckett University, Leeds, UK; 6 Norfolk and Waveney ICS, Norwich, Norfolk, UK; 7 Norwich Medical School, University of East Anglia, Norwich, Norfolk, UK; 8 Nursing and Midwifery, University of the West of England, Bristol, UK; 9 Faculty of Environment and Life Sciences, University of Southampton, Southampton, UK

**Keywords:** PAIN MANAGEMENT, REHABILITATION MEDICINE, Randomised Controlled Trial, QUALITATIVE RESEARCH, Caregivers

## Abstract

**Objectives:**

To assess the feasibility of conducting a pragmatic, multicentre randomised controlled trial (RCT) to test the clinical and cost-effectiveness of a pain management training intervention to support people with persistent musculoskeletal pain and their informal carers.

**Design:**

Two-arm, multicentre, pragmatic, open, feasibility RCT with embedded qualitative study.

**Setting:**

National Health Service (NHS) providers in four English hospitals.

**Participants:**

Adults receiving NHS care for persistent musculoskeletal pain and their informal carers.

**Intervention:**

Control: usual NHS care. Experimental: usual NHS care plus a carer-patient pain management training intervention (JOINT SUPPORT), comprising five, 1-hour, group-based sessions for patients and carers, delivered by trained physiotherapists or occupational therapists. Content included understanding pain, pacing, graded activity, fear avoidance, goal-setting, understanding the benefits of physical activity and medication management. This was re-enforced with a workbook. After the group-based sessions, patients and carers were supported through three telephone sessions.

**Randomisation:**

Central randomisation was computer-generated (2:1 Experimental:Control), stratified by hospital and patient-participant age (≤65 years). There was no blinding.

**Main outcome measures:**

Data collected at baseline and 3 months post-randomisation included screening logs, intervention logs, fidelity checklists and clinical outcomes on quality of life, physical and emotional outcomes, adverse events and resource use. Interviews with 14 patient-carer participants and six health professionals who delivered the intervention.

**Results:**

A total of 76 participants (38 patients; 38 carers) were enrolled. Sixty per cent (312/480) of patients screened were eligible with 12% consenting to be randomised (38/312). Fifty-four per cent (13/24) of the experimental group reached minimal compliance with the JOINT SUPPORT intervention. There was no evidence of treatment contamination. For patient-participant outcomes, within-group differences from baseline to 3 months favoured the control group when assessed by EQ-5D and Generalised Self-Efficacy total score, but favoured the intervention group when assessed by numerical rating scale pain, fatigue and Centre for Epidemiologic Studies Depression Scaletotal score. Qualitative data demonstrated the acceptability of the trial design and JOINT SUPPORT intervention with modifications to improve trial processes.

**Conclusions:**

The JOINT SUPPORT intervention was acceptable to patient-carer dyads and health professionals. Modifications to trial design, particularly enhanced recruitment strategies, are required.

**Trial registration number:**

ISRCTN78169443.

**Data availability statement:**

The data that support the findings of this study are available from the corresponding author (TS) on reasonable request. This includes access to the full protocol, anonymised participant-level dataset and statistical code.

**STRENGTHS AND LIMITATIONS OF THIS STUDY**

- Quantitative and qualitative evidence provides data to explain the feasibility successes and challenges in this study.
- Recruitment occurred over four different sociogeographical locations, indicating study acceptability across populations.
- Low loss to follow-up provided robustness of data.
- Recruitment was lower than anticipated, particularly for minority ethnic groups.
- There is unexplained variation in perspectives towards being a carer for a family member/friend who has persistent pain.

## Introduction

 Persistent musculoskeletal (bone, joint or muscle) pain is disabling. It is seen in all age groups.^1^ It encompasses conditions such as low back pain, fibromyalgia, osteoarthritis and other rheumatological diseases. It affects approximately 17 million people in the UK, with 9.1 million people living in England with long-term back pain.^2^ The UK’s National Health Service (NHS) costs to treat musculoskeletal diseases are in excess of £5 billion per year.^2^

In the UK, the NHS encourages patients to seek treatment for musculoskeletal pain from 2 to 6 weeks after onset,^3 4^ self-managing their condition during this time with analgesics and exercise. Persistent pain is commonly defined as pain lasting for 3 months or longer,^5^ although persistent pain trajectories have been reported to be first established 6 weeks from the onset of pain.^6^ People with persistent musculoskeletal pain frequently have difficulty managing their symptoms and maintaining independence and quality of life.^7^ To assist with this, they often access support. This may include help in tasks such as washing and dressing, preparing meals and assistance in feeding, housework or shopping.^8 9^ Such support may be ‘formal’ or ‘informal’. Formal care is defined as the provision of care by someone who is paid. Informal care is provided withoutdirect payment, often given by a spouse or partner, family members and/or friends.^10^ Being a carer for someone with persistent musculoskeletal pain can be a physical, emotional and economic burdensome.^11 12^ However, it may also be perceived by some as rewarding, worthwhile and a pleasure, providing an opportunity for the carer and the person with persistent pain to develop a new relationship together through caregiving.^13^

Usual NHS care for persistent musculoskeletal pain is focused on the patient, providing interventions to support long-term symptom management. These are either through structured programmes such as the Enabling Self-management and Coping with Arthritic Pain using Exercise-Pain programme^14^ or based on guidelines incorporating elements of education, exercise, pain relief and psychological interventions.^5 15 16^ In both instances, none of these approaches routinely include carer training to support symptom management and improve carer quality of life. Accordingly, there is a need to test a patient-carer training intervention to address this clinical challenge, both for patients and people who support them as informal carers. The purpose of this feasibility study was to test the deliverability and acceptability of such a carer-patient training intervention, and to evaluate the feasibility of a randomised controlled trial (RCT) design investigating the effectiveness of the carer-patient training intervention.

This study aimed to assess the feasibility of conducting a pragmatic, multicentre, RCT to test the clinical and cost-effectiveness of an informal carer-patient pain management training intervention to support people with persistent musculoskeletal pain.

## Methods

The study was reported to satisfy the Consolidated Standards of Reporting Trials extension for reporting pilot and feasibility RCTs.^17^ A full protocol has been published previously.^18^

### Study design

**Figure 1:**
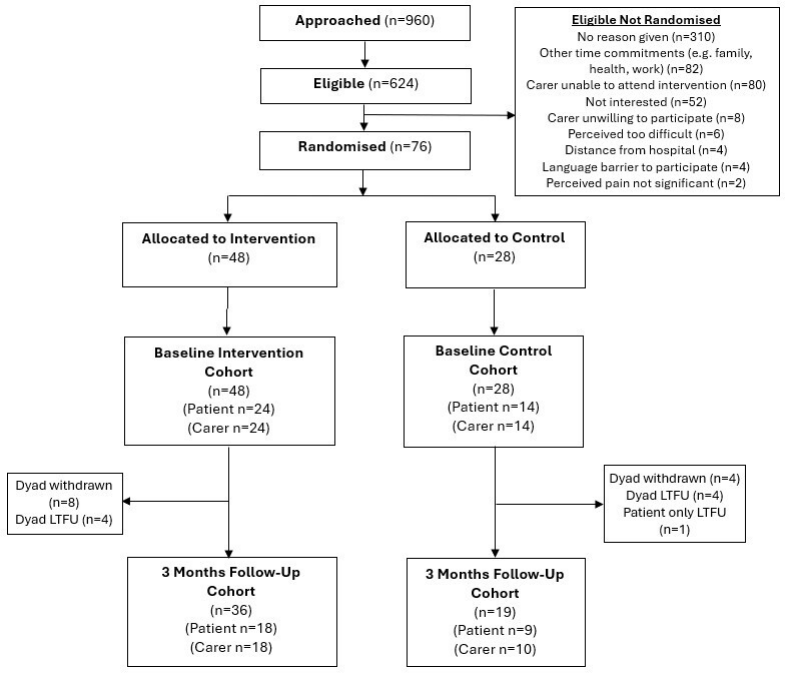
JOINT SUPPORT participant flow chart.

This was a parallel, multicentre, pragmatic feasibility RCT and embedded qualitative study. The study flow chart is presented in figure 1.

### Eligibility criteria

#### Inclusion criteria

- Patients aged 18 years and over with a history (6 weeks or more) of pain from a musculoskeletal (bone, joint or muscle) origin who were referral to, or attended physiotherapy, rheumatology, orthopaedic, occupational therapy or pain management services. Six weeks was selected as persistent pain trajectories have been reported as first established at this time point.^6^
- Patients who were able to nominate an informal carer. An informal carer was defined as an individual who had, or was expected to provide unpaid care, assistance, support or supervision in activities of daily living for at least 3 hours per week, over two or more personal contacts.
- Patients and carers willing and able to provide consent.
- Patients and carers who can engage in a group-based intervention currently delivered in English.

#### Exclusion criteria

- Patients or carers with acute (requiring hospitalisation) or terminal illness (life expectancy <6 weeks).
- Patients or carers with cognitive impairment (Abbreviated Mental Test Score <8^19^).

### Study treatments

Usual care was standard NHS treatment as usual, for example, physiotherapy, orthopaedic or pain management services. Patient-participants and their carers did not receive the JOINT SUPPORT intervention or any carer training.

The JOINT SUPPORT intervention has been previously described^18^ and is presented in accordance with the TIDieR reporting checklist^20^ (online supplemental file 1). In brief, this was a carer-patient dyad pain management training intervention which was developed by patients, carers and health professionals. The theoretical principle behind the intervention is social learning theory.^21^ People randomised to the experimental group received the usual NHS care with the addition of the JOINT SUPPORT intervention. The only difference between the groups was the addition of five, 60 min, group-based, health professional-carer dyad JOINT SUPPORT training sessions, and three follow-up telephone calls 1, 3 and 6 weeks after the final group session. In the outpatient sessions, participants were taught skills in understanding pain, pacing, graded activity, fear avoidance, goal-setting, benefits of physical activity and medication management. This was re-enforced with a workbook. The follow-up telephone calls by health professionals aimed to re-enforce the skills developed in the group-based sessions, support any setbacks in recovery and develop longer-term goals.

Each health professional (physiotherapist or occupational therapist) who delivered the experimental intervention attended a half-day JOINT SUPPORT training session, delivered by the research team, which taught the components and format of the intervention.

### Data collection

At the time of screening, sites verified participants’ eligibility and recorded anonymised demographic characteristics in the screening log. Baseline assessments were undertaken after consent was obtained, prior to randomisation. Data collected at baseline included: for patient- and carer-participant: age, sex, ethnicity, occupational status (current or past if retired), medical comorbidities, presenting musculoskeletal pathology(ies); for patient-participant: duration of symptoms, the relationship of carer to the patient; and for carer-participant: duration of caring, whether a concurrent carer for another person, whether living with patient-participant.

Participants were followed up at 3 months post-randomisation. Data were collected via postal questionnaires by the central trial team.

### Outcome measures

To answer our feasibility objectives, we assessed the feasibility of recruitment using screening log data on the number of potential patients screened, assessed for eligibility, including reasons for exclusion/non-participation, and consented. We assessed intervention acceptability through qualitative interviews, in addition to recording study attrition and analysing acceptability questionnaires.^22^ We assessed the intervention fidelity of the healthcare professional, using intervention log checklist data on intervention timing, duration and frequency, as well as qualitative interview responses. We assessed the intervention fidelity of carers through examination of the carer intervention logs. Randomisation acceptability was assessed through examination of site screening logs, eligibility assessment logs and consent forms, in addition to monitoring participant attrition between the groups and responses in qualitative interviews. We assessed the risk of contamination by examining intervention log data including experimental and control intervention records, QA monitoring visit checklists, delegation logs and responses in the qualitative interviews. Completeness of outcome measures was assessed through completion rates (baseline and 3 months post-randomisation). Outcome measures collected are illustrated in online supplemental file 2. Finally, a signal of effectiveness was determined by reporting effect size of the clinical measures listed in online supplemental file 2.

### Randomisation and blinding

Randomisation was stratified at the individual-dyad level (2:1 Experimental: Control) by hospital and age of patient-participant (< or ≥65 years). The central trial team members performed randomisation post-baseline data collection. Allocation was concealed to participants and health professionals prior to randomisation. Randomisation was computer-generated on a secure, online programme, centrally administered by a programmer at the Norwich CTU (NCTU). The randomisation sequence was generated by NCTU programmers and tested by the trial statistician.

Due to the participatory nature of the intervention, blinding participants or the site team was deemed impractical in this feasibility study.

### Sample size

We aimed to recruit 160 participants (80 patients; 80 carer-participants) to have outcome data on 140 participants (70 patients; 70 carer-participants) after drop-out, based on Teare *et al*’s^23^ recommendations. Based on that suggestion, this sample size was considered sufficient to answer our feasibility objectives and assess the a priori progression criteria.^18^ The sample size also allowed each site to deliver the JOINT SUPPORT intervention in at least two complete cycles to determine deliverability and acceptability across study sites.

### Data analysis and progression criteria

Consent rates, recruitment rates, attrition, missing data rates and intervention fidelity were reported as proportions, with 95% CI presented for consent and recruitment rates. The analysis of clinical outcome measures was descriptive, reported as medians and IQR and frequency and percentages for binary and categorical variables, respectively. Since this was a feasibility study and was not a fully powered trial, no formal statistical testing was undertaken. This was as planned in the protocol.^18^ A ‘traffic light’ system was used as a guide for progression to a definitive trial,^24^ centred around recruitment, retention, intervention fidelity, contamination and data collection.

Intervention adherence was assessed as full compliance, minimal compliance or non-compliance. Full compliance equated to attendance in five group sessions and three telephone calls. Minimal compliance was defined as attendance to three or four group sessions (with the requirement of attending Session 1 or 2) and one (or more) telephone call(s). Minimal compliance would also include those attending five group sessions plus one or two telephone calls. Non-compliance was defined as attendance at two or fewer (one or two) group sessions irrespective of the number of telephone calls. Attendance required both members of the dyad in each. This was presented as a percentage and 95% CI using the Clopper-Pearson exact method due to a small sample size or proportion close to 0 or 1.

### Study monitoring

A Trial Oversight Committee (TOC) was appointed to independently review data on safety, protocol adherence and study processes.

### Patient and public involvement and engagement

Patient involvement began during protocol development and continued throughout the study. One patient-member (not enrolled in the study) attended Trial Management Group meetings and one member (not enrolled in the study) was a member of the TOC. They provided insights into study conduct, particularly on data collection processes, and helped interpret the findings. Participants who expressed an interest in receiving information on the findings were provided with this at the end of the study.

### Embedded qualitative study

The embedded qualitative study explored the acceptability of the research design and the JOINT SUPPORT intervention from the perspective of carer-dyads and health professionals. Our approach and interview topic guide were informed by the Medical Research Council (MRC) guidance for evaluating complex interventions and included one member of the public who had lived experience of persistent musculoskeletal pain.^25^,^27^

Up to 6 weeks post-intervention, carer-dyads were invited to participate in an in-depth, semistructured interview. They were purposively sampled by study site, age, ethnicity, duration and severity of disability (measured by baseline Musculoskeletal Health Questionnaire)^28^ and the duration the carer had been caring for the patient-participant. Health professionals who had been involved in the study set-up, participant recruitment and/or the delivery of the intervention were also invited to be interviewed and were purposively sampled to ensure representation across study sites.

All interviews were conducted by the same researcher (AW), an experienced postdoctoral, female, qualitative researcher. AW had no role in study recruitment or intervention delivery. Interviews were undertaken via telephone or Microsoft Teams and were audio recorded, transcribed verbatim and anonymised.

Data were analysed taking a two-stage approach aligned to the MRC framework on process evaluations^26 27^ to understand the context, mechanism and fidelity of the intervention implementation. We initially analyse all data deductively to assess the quality of implementation and clarify the hypothesised causal mechanisms identified in our logic model, identifying contextual factors associated with variation in outcomes and how the intervention might be optimised for acceptability. Second, we analysed the data more inductively, critiquing the conceptual approach of the JOINT SUPPORT intervention, understanding any unintended consequences and reflections on the intervention from the healthcare professional, patient and carer perspective.

Data analyses were led by AW, with a discussion with SH and TS to ensure rigour in our interpretations of the data. Data from both participants and health professionals were synthesised to allow for perspectives of the intervention to be reported via the different lens, exploring the holistic and nuanced experiences of the JOINT SUPPORT intervention.

## Results

### Participant characteristics

We recruited 76 participants (38 patients; 38 carers) from November 2022 to October 2023 (figure 1). Forty-eight participants were randomised to the experimental group, and 28 to the control group.

A summary of the patient-cohort characteristics is presented in table 1. Seventy-nine per cent (30/38) were female, with a mean age of 52.5 years in the intervention group and 41.5 years in the control group. In total, 66% (25/38) were white British or Irish. A summary of the carer-cohort characteristics is presented in table 2. Forty-five per cent (17/38) were female, with a mean age of 60.5 years in the intervention group and 53.5 years in the control group. Fifty per cent of the carer-cohort was in paid employment.

**Table 1:** Descriptive statistics of patient-participant characteristics at baseline

	InterventionN=24	ControlN=14
Gender: n (%)		
Male	4 (16.7)	3 (21.4)
Female	19 (79.2)	11 (78.6)
Self identify as ‘non-binary’	1 (4.2)	0
Age in years: median (IQR)	52.5 (33.0, 69.5)	41.5 (36.0, 60.0)
Age group: n (%)		
≥65 years	8 (33.3)	3 (21.4)
<65 years	16 (66.7)	11 (78.6)
Ethnicity: n (%)		
White British	18 (78.3)	7 (50.0)
White other	1 (4.4)	3 (21.4)
White and Black Caribbean	0	1 (7.1)
White and Black African	1 (4.4)	1 (7.1)
White and Asian	1 (4.4)	0
Indian	1 (4.4)	0
Asian – other	0	1 (7.1)
Other	1 (4.4)	1 (7.1)
Missing	1	0
Height (cm): median (IQR)	160.0 (157.5, 170.2)	163.0 (160.0, 172.0)
Missing	1	1
Weight (kg): median (IQR)	74.5 (59.0, 80.0)	76.0 (67.6, 90.0)
Missing	2	1
BMI: median (IQR)	28.1 (22.3, 31.3)	27.9 (24.3, 32.7)
Missing	2	2
Musculoskeletal pathology:* n (%)		
Fracture	4 (16.7)	5 (35.7)
Inflammatory arthritis	8 (33.3)	1 (7.1)
Joint replacement	2 (8.3)	1 (7.1)
Fibromyalgia	7 (29.2)	5 (35.7)
Hypermobility	7 (29.2)	4 (28.6)
Low back pain	13 (54.2)	10 (71.4)
Tendinopathy	2 (8.3)	2 (14.3)
Joint dislocation/instability	8 (33.3)	5 (35.7)
Osteoarthritis	12 (50.0)	1 (7.1)
Rheumatoid arthritis	4 (16.7)	0
Other†	7 (29.2)	5 (35.7)
Pain location:* n (%)		
Spinal pain	21 (87.5)	10 (71.4)
Shoulder	15 (62.5)	9 (64.3)
Elbow	10 (41.7)	4 (28.6)
Wrist	12 (50.0)	8 (57.1)
Hand	14 (58.3)	7 (50.0)
Hip	17 (70.8)	6 (42.9)
Knee	14 (58.3)	11 (78.6)
Ankle	10 (41.7)	7 (50.0)
Foot	13 (54.2)	7 (50.0)
Other	9 (37.5)	3 (21.4)
Musculoskeletal pathology symptoms:* n (%)		
Pain	24 (100.0)	14 (100.0)
Fatigue	22 (91.7)	11 (78.6)
Stiffness	20 (83.3)	11 (78.6)
Reduced concentration	18 (75.0)	9 (64.3)
Anxiety	13 (54.2)	6 (42.9)
Depression	11 (45.8)	7 (50.0)
Other‡	6 (25.0)	4 (28.6)
Duration of symptoms (months): median (IQR)	36 (13, 96)	61 (24, 96)
Missing	6	1
Previous healthcare interventions: n (%)		
Yes	15 (93.8)	10 (83.3)
No	1 (6.3)	2 (16.7)
Missing	8	2
Past medical history:* n (%)		
Cardiac condition (heart)	1 (4.2)	2 (14.3)
Asthma	4 (16.7)	3 (21.4)
COPD	0	0
Hypertension	6 (25.0)	3 (21.4)
Diabetes (type 1 or 2)	2 (8.3)	1 (7.1)
Stroke	1 (4.2)	2 (14.3)
Cancer	2 (8.3)	0
Osteoarthritis	12 (50.0)	1 (7.1)
Low back pain	14 (58.3)	9 (64.3)
Depression	10 (41.7)	6 (42.9)
Anxiety	11 (45.8)	7 (50.0)
Dementia	0	0
Other§	13 (54.2)	8 (57.1)
Occupational status: n (%)		
Unemployed	1 (4.2)	2 (14.3)
Paid employment (full or part-time)	6 (25.0)	6 (42.9)
Retired	6 (25.0)	3 (21.4)
Unable to work	11 (45.8)	3 (21.4)

* Percentages are those that responded ‘yes’ to each separate item.

† Other musculoskeletal pathology includes: Sstill recovering from meniscus repair surgery left knee, under investigation, scoliosis, osteonomy, TMJ/oral and maxillofacial, and tendon rupture.

‡ Other musculoskeletal pathology symptoms include: spasms, constipation, insomnia/hypersomnia, orthostatic intolerance, seating, just get fed up, nausea, loss of appetite, swelling, tinnitus, balance issues, sleep problems, and brain fog.

§ Other past medical history includes: hemiplegic migraine, deafness, hysterectomy, asplenic psoriatic arthritis, POTS, MCAS, IBS interstitial cyctitus, costochondritlis PTSD, hypermobile Ehlers-Danlos, endometriosis, hiatus hernia, rectocele bladder issues, perrennial allergic rhinitis, thalassemia trait, dyslexia, delayed sleep rhythm, insomnia, hypersombnia, borderline personality disorder, ADHD, frontal lobe brain damage, cellulitis, leg ulcers, BRCA2 gene, FND, chronic allergic rhinitis, rheumatoid/inflammatory arthritis, migraines, systemic sclerosis, underactive thyroid, MS, thyroiditis, Reaynaud’s, gastritis, plantar fasciitis, AF, CRPS, EDS, psoriasis, and Graves’ disease.

BMI, body mass index; COPD, chronic obstructive pulmonary disease; n, number of participants.

**Table 2:** Descriptive statistics of carer-participant characteristics at baseline

	Intervention	Control
	n=24	n=14
Gender: n (%)		
Male	14 (58.3)	7 (50.0)
Female	10 (41.7)	7 (50.0)
Age in years: median (IQR)	60.5 (47.5, 72.0)	53.5 (37.0, 61.0)
Ethnicity: n (%)		
White British	18 (75.0)	8 (61.5)
White other	1 (4.2)	2 (15.4)
White and Black Caribbean	0	1 (7.7)
White and Black African	1 (4.2)	0
Indian	1 (4.2)	0
Asian – other	0	1 (7.7)
Black - other	1 (4.2)	0
Other	2 (8.3)	1 (7.7)
Missing	0	1
Height (cm): median (IQR)	171.5 (165.0, 177.8)	170.2 (167.6, 175.3)
Missing	1	1
Weight (kg): median (IQR)	80.7 (70.8, 95.3)	95.0 (77.1, 111.1)
Missing	1	2
BMI: median (IQR)	28.5 (25.1, 31.2)	31.7 (26.6, 37.0)
Missing	1	2
Musculoskeletal pathology:* n (%)		
Fracture	3 (12.5)	3 (21.4)
Inflammatory arthritis	3 (12.5)	5 (35.7)
Joint replacement	0	2 (14.3)
Fibromyalgia	1 (4.2)	1 (7.1)
Hypermobility	2 (8.3)	1 (7.1)
Low back pain	11 (45.8)	9 (64.3)
Tendinopathy	0	1 (7.1)
Joint dislocation/instability	1 (4.2)	1 (7.1)
Osteoarthritis	4 (16.7)	3 (21.4)
Rheumatoid arthritis	3 (12.5)	0
Other†	2 (8.3)	3 (21.4)
Symptoms locationa:* n (%)		
Spinal pain	12 (50.0)	7 (50.0)
Shoulder	7 (29.2)	1 (7.1)
Elbow	2 (8.3)	2 (14.3)
Wrist	2 (8.3)	3 (21.4)
Hand	4 (16.7)	7 (50.0)
Hip	4 (16.7)	3 (21.4)
Knee	8 (33.3)	7 (50.0)
Ankle	2 (8.3)	3 (21.4)
Foot	4 (16.7)	4 (28.6)
Other	1 (4.2)	1 (7.1)
Musculoskeletal pathology symptoms*: n (%)		
Pain	15 (62.5)	8 (57.1)
Fatigue	7 (29.2)	4 (28.6)
Stiffness	11 (45.8)	8 (57.1)
Reduced concentration	4 (16.7)	3 (21.4)
Anxiety	5 (20.8)	4 (28.6)
Depression	2 (8.3)	3 (21.4)
Other‡	2 (8.3)	2 (14.3)
Duration of symptoms (months): median (IQR)	24 (12, 72)	98 (48, 120)
Missing	13	4
Past medical history*: n (%)		
Cardiac condition (heart)	2 (8.3)	3 (21.4)
Asthma	2 (8.3)	3 (21.4)
COPD	0	0
Hypertension	2 (8.3)	2 (14.3)
Diabetes (type 1 or 2)	4 (16.7)	1 (7.1)
Stroke	1 (4.2)	1 (7.1)
Cancer	3 (12.5)	0
Osteoarthritis	3 (12.5)	2 (14.3)
Low back pain	8 (33.3)	7 (50.0)
Depression	6 (25.0)	5 (35.7)
Anxiety	6 (25.0)	7 (50.0)
Dementia	0	0
Other§	1 (4.2)	2 (14.3)
Occupational status: n (%)		
Unemployed	2 (8.3)	1 (7.1)
Paid employment (full or part-time)	11 (45.8)	8 (57.1)
Retired	9 (37.5)	3 (21.4)
Unable to work	2 (8.3)	2 (14.3)
Relationship to patient: n (%)		
Spouse	11 (47.8)	8 (57.1)
Partner	3 (13.0)	2 (14.3)
Daughter/Son	2 (8.7)	2 (14.3)
Parent	7 (30.4)	2 (14.3)
Missing	1	0
Residential status - living with patient: n (%)		
Yes	18 (75.0)	11 (78.6)
No	6 (25.0)	3 (21.4)
Previously caregiver for this patient: n (%)		
Yes	17 (70.8)	7 (53.9)
No	7 (29.2)	6 (46.2)
Missing	0	1
Previously caregiver for another patient: n (%)		
Yes	7 (29.2)	2 (16.7)
No	17 (70.8)	10 (83.3)
Missing	0	2

* Percentages are those that responded ‘yes’ to each separate item.

† Other musculoskeletal pathology includes: chondral lesion left shoulder, osteopenia, joint pain, and CRPS.

‡ Other musculoskeletal pathology symptoms include: pins and needles numbness, anaemia, spasms, and numbness of right-hand fingers.

§ Other past medical history includes: hereditary hemorrhagichaemorrhagic telangiectasia (), hypothyroidism, chronic anaemia, cholesterol, borderline diabetic, PoTs and EDS (hypermobility type).

BMI, body mass index; COPD, chronic obstructive pulmonary disease; n, number of participants.

Online supplemental file 3 illustrates the health services accessed by patient-participants during the study, as reported by the site team. Physiotherapy, GP and pain services were most frequently used (78%, 26% and 26%, respectively).

### Feasibility outcomes

The outcomes of the progression criteria traffic-light assessment are presented in table 3.

**Table 3:** Outcomes of the progression criteria traffic-light assessment

	Green (Go)	Amber (Amend)	Red (Stop)
Recruitment	>30% of the patients screened across the sites in 12 months would be eligible	20%–30% would be eligible	<20% would be eligible
Intervention fidelity*	>70% of the participant-dyads compliant with their allocated intervention as randomised	50%–70% received intervention as randomised	<50% received intervention as randomised
Randomisation acceptability	>40% of the eligible participants consent to be randomised	20%–40% would be randomised	<20% would be randomised
Contamination	<5% of the participants in either group received majority of their allocated treatment cross-over	5%–10% of the participants cross-over	>10% of the participants cross-over
Data collection completion	<15% missing outcome questionnaires for whatever reason at 3-month data collection	15%–30% missing questionnaires	>30% missing questionnaires

* defined as mMinimum compliance to JOINT SUPPORT intervention receipt.

### Recruitment, retention and randomisation acceptability

In total, 480 dyads (n=960) were screened with 312 dyads (n=624) eligible (65%; 95% CI: 60.6 to 69.3). Thirty-eight dyads (n=76) consented who were eligible (12%; 95% CI: 8.8 to 16.3). The most frequent reasons for eligible participants not wishing to participate (when stated) included other time commitments (n=82) and carer unable to attend the intervention sessions (n=80) (figure 1).

At 3-month follow-up, 26 patient-participants (71.1%; 95% CI: 54.1 to 84.6) and 28 carer-participants (73.7%; 95% CI: 56.9 to 86.6) remained in the study.

### Intervention fidelity (health professionals)

Online supplemental file 4 illustrates the delivery of JOINT SUPPORT. In total, 18 participant-dyads out of 24 (75%) allocated to the JOINT SUPPORT intervention received the intervention. Online supplemental file 4 demonstrates all components of the intervention were received by participants, with pacing, graded-activity, goal-setting, medication use and pain education consistently delivered.

Intervention full compliance was reported in 21% (95% CI: 7.1 to 42.2) of the JOINT SUPPORT cohort (5/24), and minimal compliance was reported in 54% (95% CI: 32.8 to 74.5). Online supplemental file 5 illustrates the frequency to which one member of the dyad compared with both members of the dyad attended each JOINT SUPPORT session. Attendance by both members of the dyad for the face-to-face sessions ranged from 58% to 71%, while the range was 63% to 79% for one member of the dyad. A higher proportion of both members of the dyad were in attendance for the telephone calls (range: 63% to 79%) (online supplemental file 5).

### Intervention fidelity (carers)

Only one carer-participant returned their carer log. Accordingly, there were insufficient data to permit the assessment of intervention fidelity. This was therefore not analysed.

### Contamination

From the qualitative investigations, case report forms for treatment received, protocol deviation reports and delegation logs of treating health professionals, there was no evidence of between-group intervention contamination.

### Outcome data response rates

Online supplemental file 6 illustrates data completeness at baseline and 3-month follow-up for carer-patient participants. For patient-participants, all baseline and 3-month follow-up questionnaires had a response rate of above 88%, with the exception Generalised Self-Efficacy (GSE) total score (78% 3-month control group). All carer-participant baseline and 3-month follow-up questionnaires had a response rate of 88% or above.

### Clinical outcomes

The descriptive analysis for clinical outcomes is presented in online supplemental file 7. This should be interpreted with caution given the underpowered cohort and baseline differences.

For patient-participant outcomes, within-group difference outcomes from baseline to 3 months favoured the control group when assessed by EQ-5D and GSE total score, but favoured the intervention group when assessed by numerical rating scale pain, fatigue and Centre for Epidemiologic Studies Depression Scale (CES-D) total score (online supplemental file 7).

For carer-participant outcomes, within-group difference outcomes favoured the control group when assessed by CES-D total score and Leisure Time Satisfaction but favoured the intervention arm when assessed by EQ-5D visual analogue scale and the Zarit Burden Score (online supplemental file 7).

No participant, from either group, reported a related adverse event or serious adverse event.

Intervention acceptability questionnaire data indicated that the JOINT SUPPORT intervention was considered acceptable by patient- (online supplemental file 8) and carer-participants (online supplemental file 9).

### Qualitative results

**Figure 2:**
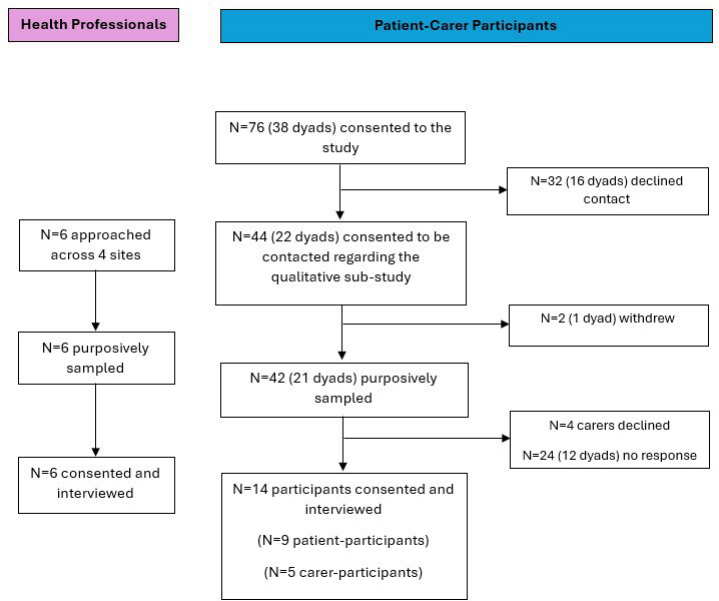
Qualitative substudy participant flow chart.

Fourteen carer-patient and six health professional interviews took place between September 2023 and March 2024. Figure 2 is a flow chart of carer-patient dyad recruitment to the qualitative substudy. Participant characteristics are summarised in table 4. The mean duration of interviews was 42.6 min (range: 21.5 to 74.6 min).

**Table 4:** Interview study participant characteristics (patient, carer and health professional)

Patient-Participants (n=9)	
Gender (n, %)	
Male	1 (11.1)
Female	8 (88.9)
Age (years)	
(mean, SD)	59.7 (15.4)
(median, IQR)	54.0 (50.0, 74.0)
Ethnicity (n, %)	
White British	8 (88.9)
White and Asian	1 (11.1)
Occupation (n, %)	
Unemployed	1 (11.1)
Paid employment	2 (22.2)
Retired	3 (33.3)
Unable to work	3 (33.3)
Musculoskeletal condition* (n, %)	
Fracture	2 (22.2)
Inflammatory	3 (33.3)
arthritis	1 (11.1)
Joint replacement	3 (33.3)
Fibromyalgia	1 (11.1)
Hypermobility	4 (44.4)
Low back pain	1 (11.1)
Joint dislocation/instability	3 (33.3)
Osteoarthritis	1 (11.1)
Rheumatoid arthritis	2 (22.2)
Other†	
Time with condition, months	
(mean, SD)	35.3 (27.5)
(median, IQR)‡	36 (12, 38)
Missing	3
Site (n, %)	
Site 1	1 (11.1)
Site 2	5 (55.6)
Site 3	3 (33.3)
Carer-Participants (n=5)	
Gender (n, %)	
Male	3 (60.0)
Female	2 (40.0)
Age (years)	
(mean, SD)	57.4 (16.8)
(median, IQR)	48.0 (47.0, 74.0)
Ethnicity (n, %)	
White British	4 (80.0)
White Other	1 (20.0)
Occupation (n, %)	
Paid employment	3 (60.0)
Retired	2 (40.0)
Living with patient (n, %)	
Yes	4 (80.0)
No	1 (20.0)
Previous carer for this patient (n, %)	
Yes	4 (80.0)
No	1 (20.0)
Previous carer for another patient (n, %)	
Yes	1 (20.0)
No	4 (80.0)
Site (n, %)	
Site 2	3 (60.0)
Site 3	2 (40.0)
Health Professional-Participants (N=6)	
Job role (n, %)	
Physiotherapist	6 (100.0)
Site (n, %)	
Site 1	3 (50.0)
Site 2	1 (16.6)
Siten 3	1 (16.6)
Site 4	1 (16.6)

* Percentages are those thatwho responded ‘yes’ to each separate item.

† Other MSK conditions includes scoliosis and missing other information=1.

‡ Not given if n<3.

Findings are grouped around the core components of a process evaluation.^26 27^

### Contextual factors in the study design

Among health professionals, the recruitment process dominated. Challenges included the initial approach to dyads, as often, carers were absent in the outpatient appointments when introducing the study. Additionally, there was an apparent lack of communication among dyads about participating in the research, so health professionals could not rely on potential patient-participants to reliably share study information with carers. Such challenges in approaching both members of the dyad were exacerbated by reported health professionals’ time pressures and clinical priorities.

Health professionals suggested the most common reasons for dyads’ declining participation were the significant time commitment, competing responsibilities and saturation of hospital appointment times.

It’s often been when the carer has been very busy. When the carer is doing a lot for that individual, they don’t have the time to then do this other thing [JOINT SUPPORT], That’s been a bit of a barrier [to recruitment]. (Physiotherapist, Site 3)

Some dyads perceived JOINT SUPPORT to offer an opportunity to access further support and try new approaches to pain management. Health professionals perceived involvement as a learning opportunity for carers, as patient-participants wanted their carers to better understand their condition and as an opportunity to facilitate effective communication among dyads and share advice.

[They join to] get a bit of help, and they want their caregivers to attempt to do joint support so that they can understand more about why they need a bit more care and support. (Physiotherapist 3, Site 2)

The opportunity to meet and talk to people with a shared experience of pain was also valued by participants and contributed to the reasons for participating.

I thought it would be nice to meet somebody with the same things [pain] and talk. Talk the condition over, it might be nice to listen to other people. (Patient-participant, Female, Employed, Site 3, Intervention)

In terms of outcome measures, participants’ perceptions were generally positive, being easy to complete. Health professionals found them relevant to their practice. Some carers were mindful of the privacy needed to complete the questionnaires, which raises questions about carers’ ability to share honest thoughts and self-reflection.

With the initial questionnaire, I did think it was quite funny because you're sitting next to each other to do it and there’s some stuff like, ‘Does it make you resent the person that you're caring for?’ and I thought, Jesus, I hope people aren’t at home looking over each other’s shoulders! (Carer-participant, Male, Son, Employed, Site 2, Intervention)

### Fidelity of the intervention

#### Training

In general, health professionals felt well-prepared to deliver the intervention. One physiotherapist recommended the use of patient advisors along with the trial team to facilitate the sessions and model the scenarios. Health professionals felt this could improve their competence in communication and approaching more sensitive topics.

Some of that could be delivered by one of the patient and public involvement members participants or patient advisors. I wonder whether that could have been more engaging for staff. That could have helped us in terms of how we communicate some of the topics as well. (Physiotherapist 3, Site 2)

#### Experience of the intervention

Participants’ experience of participating in JOINT SUPPORT was largely positive. Carers saw the value in participating. They acknowledged the benefits it had for patients. Carers were grateful for the advice shared among the groups and perceived there to be improvements as a result. However, the travel and time burden of several face-to-face sessions was challenging, particularly among those who were employed.

Sessions could have been a bit later in the morning, or maybe it should be in the afternoon to give people more time in the morning because if you’ve got certain conditions, it’s a struggle in the morning if you've got lots of pain or if you’ve got to come from somewhere. (Patient-participant, Female, Employed, Site 2, Intervention)

#### Group sessions

Overall, participants shared their enjoyment of the sessions and how they valued the opportunity for socialising. Dyads appreciated building rapport with others in the group, enabling honest and open conversations about their experiences of living with pain or caring for someone with pain. The shared experience allowed feelings and experiences of living with pain to be consolidated and validated and a sense of comfort, and even gratitude, for those who perceived others to be faring worse than themselves.

Yeah, it’s quite nice to remind yourself that people are in worse situations. There are people in similar situations and worse situations as well because, not in a horrible way, it makes you count your blessings, doesn’t it? (Carer-participant, Male, Employed, Site 2, Intervention)

Some participants reported a preference for ‘condition-specific’ groups, as the comparison to others felt unhelpful. Some felt false hope around the management of pain and recovery time—this was particularly present for those who were living with chronic, progressive conditions when compared with those postsurgery.

#### Workbook

Health professionals reflected on the design of the workbook and, in some places, found it difficult to pitch the right content to the right audience (ie, patient-participant vs carer-participant), particularly when referring to goal-setting activities.

Although participants shared that the workbooks were beneficial in sharing knowledge about pain management, the case studies/scenarios did not resonate with people with pain. It may have been that the broad inclusion criteria and the variety of needs across the group meant that people did not identify with some of the content and scenarios provided. Others, however, saw the value in conversations about the scenarios and problem-solving as a team, creating a sense of empowerment.

The case studies just felt really powerful. I thought the way that people were giving advice to made-up people was really powerful because people actually started to talk about themselves and talk about their relationship with the other person. I thought that worked really well. (Physiotherapist, Site 3)

#### Follow-up telephone calls

The JOINT SUPPORT telephone calls were reported by dyads to be a helpful and easy process. Participants valued health professionals’ time and empathy and felt comfortable asking further questions. The views of health professionals resonated with this. Follow-up telephone calls were seen as an opportunity to address additional needs by signposting people to other support or services.

### Mechanisms: factors that support or impede the implementation

Barriers, facilitators and suggested improvements to the JOINT SUPPORT study, as suggested by participants (patients, carers and health professionals), are extensively detailed in online supplemental file 10.

## Discussion

The results of this study indicate feasibility in the identification of potential participants for an effective trial of JOINT SUPPORT and low risk of intervention contamination. However, further study modifications are required to improve intervention fidelity and conversion of eligible participants to enrolments and in data collection processes. The qualitative study highlights the acceptability of the JOINT SUPPORT intervention, offering perceived potential benefit by patients, carers and physiotherapists, although with suggested improvements. It also highlights the challenges in study design and participation, particularly against a backdrop of work and social time constraints when patients and carers are relatively young with occupational and social implications.

The study’s inclusion criteria were broad, including people with persistent musculoskeletal pain seeking NHS care, irrespective of diagnosis. This was justified given the potential treatment effect which the JOINT SUPPORT intervention proposed to offer, not targeting a specific pathology which may have warranted more selective eligibility criteria. This was reflected in the high proportion of eligible participants screening. However, the qualitative findings indicated a preference by some physiotherapists for a more specific patient population, allowing them to ‘target’ study screening to a patient-population. A future study should consider whether there is a specific population of most ‘need’ or most receptive to the JOINT SUPPORT intervention, which may offer a more targeted approach to recruitment.

The JOINT SUPPORT intervention was delivered face-to-face in three of the four sites for the majority of intervention participant-dyads. Based on the screening data, a major reason why participants did not wish to enrol in the study was the inability to attend a hospital appointment. Given over half the cohort was in part-time or full-time work, managing competing interests on time could be challenging. One site offered the JOINT SUPPORT intervention online but there appeared no clear distinction in recruitment or retention differences, nor in the characteristics of those who were recruited to that site over the other three sites, while acknowledging the small numbers recruited per site. There remain issues regarding the possibility of digital exclusion when solely offering interventions online.^29^ Nonetheless, this may be one strategy to overcome the low conversion of eligible-to-randomised participants, while also opening a potentially beneficial intervention to people who may struggle to be able to attend and engage with some or all of the sessions. Further consideration of mode and timing of intervention delivery is warranted.

Previous literature has highlighted the tension that some carer-participants faced in being identified as a carer or not.^13^ Carer-participants frequently reported considering themselves as a ‘support’ but not an individual’s ‘carer’. This may explain why some eligible carers did not wish to participate, believing it was not relevant to them, or potential patient-participants were unable to identify a nominated carer. It may also provide some explanation for the low intervention compliance and compliance in returning carer-specific data, particularly the carer intervention log. Nomenclature to describe an informal carer is varied, having been referred to as ‘informal caregivers’,^11^ ‘unpaid caregiver’,^7^ ‘key support’^30^ or ‘buddy’.^31^ While this study used the term informal carer, the findings suggest that this may not necessarily be the correct term for supporters of people with persistent pain and therefore further consideration with patients, their family members and friends, clinicians, patient-groups or associated charities may be valuable to establish a more appropriate ‘term’ to describe these individuals.

This feasibility study was not powered to assess the effectiveness of the carer-patient training intervention. The results indicated some promise of effect, when analysed descriptively for outcomes including pain, fatigue and depression for patient-participants and carer burden and health-related quality of life for the carer-participants. There has been limited evidence previously undertaken to compare these findings. Schmid *et al*’s^32^ patient-carer programme ofyoga with a self-management programme delivered to dyads also showed promise in pain perception. However, this was a pilot study. Therefore, as with the JOINT SUPPORT intervention, a further, definite trial is warranted to understand the effectiveness of such interventions.

This study has strengths and limitations. As strengths, the study recruited participants across four study sites, providing an opportunity to recruit participant-dyads across different communities. Previous literature has acknowledged limited opportunities for people from different minority ethnic groups and socioeconomic backgrounds to participate in musculoskeletal trials.^33^ Furthermore, this is the first study to provide insights on dyadic perspectives of pain management. However, limitations included the inability to recruit to target, difficulties in intervention delivery to full compliance and low conversion of eligibility participants recruited. This suggests that further consideration on both the approach and intervention delivery should be considered. The study presented with a 24% loss to follow-up during the 3-month follow-up period. This may be attributed to the burden of participating in the study for both members, particularly for those with work or family commitments. The qualitative substudy recruited physiotherapists but was unable to recruit occupational therapists. Exploring whether professional background influences perceptions towards a carer-patient training intervention for persistent pain should be considered in the future. Finally, the study design did not blind participants or sites to group allocation. This was deemed too challenging due to the participatory nature of the intervention with patient- and carer-participants, and in the delivery of these specific experimental and control groups. However, other studies have overcome such challenges with attention control participants who are blinded to receive non-therapeutic training information versus therapeutic training intervention, and those delivering this blinded in a similar way.^34^ Further exploration of the acceptability and deliverability of such approaches would be required before consideration within a full trial.

## Conclusions

While this feasibility study design demonstrates some challenges in both recruitment and intervention fidelity, the qualitative study results indicate the acceptability and potential benefit of the JOINT SUPPORT intervention perceived by people with persistent musculoskeletal pain, their carers and health professionals. Future modification to the trial design is required to ensure that this study could be feasible to robustly determine the clinical and cost-effectiveness of the JOINT SUPPORT intervention.

## Supplementary material

10.1136/bmjopen-2024-095069 online supplemental file 1

## Data Availability

Data are available upon reasonable request.
