# Supplementary material for: Pain management training for people with persistent pain and their informal carers (JOINT SUPPORT): multicentre randomised controlled feasibility trial with embedded qualitative study in English musculoskeletal services
Source: BMJ Open. 2025 Apr 15;15(4):e095069. doi: 10.1136/bmjopen-2024-095069 (PMC12001371; doi:10.1136/bmjopen-2024-095069)
Supplement: online supplemental file 1 [file bmjopen-15-4-s001.docx]

**Supplementary File 1:** JOINT SUPPORT Programme presented in accordance with the TIDieR Intervention Checklist

| **TIDieR Item** | **Description** |
| --- | --- |
| **Brief Name** | JOINT SUPPORT: patient-carer training intervention for managing persistent musculoskeletal pain. |
| **Why** | Carer-training interventions, in principle, improve the health and well-being of patients and their carers through better self-management skills. Enhancing the skills, capability, motivation, confidence and knowledge so people with persistent musculoskeletal pain can better manage their symptoms may reduce demand on health services by improving self-management. The programme is developed and informed by Social Cognitive Theory.[21] |
| **What: Materials** | Intervention Workbook are provided (paper-based) to participants (patients and carer-participants) outlining the programme and providing worksheets of activities associated with each session to re-enforce learning.  Intervention manuals and PowerPoint slides are provided as part of the training for those delivering the JOINT SUPPORT programme as part of a 3-hour training session. |
| **What: Procedures** | JOINT SUPPORT consists of 5, 1-hour group-based sessions where patients and their carers are facilitated to consider pain self-management skills to improve health outcomes. Sessions are face-to-face (3 sites) or via video-conferencing (1 site). There is a cognitive-behavioural approach used throughout the sessions, underpinned by social cognitive theory where dyads will be facilitated by a JOINT SUPPORT healthcare professional.  As outlined in the protocol paper,[18] session content includes: Session 1  - Understanding pain, caregiving and how pain affects the caregiving dyad. - Introduction and explanation of the JOINT SUPPORT Workbook, highlighting material on pain and effects on the dyad.  Session 2  - Pacing and graded activity. - Goal setting. - JOINT SUPPORT Workbook—highlighting material on goal-setting and problem-solving.  Session 3  - Benefits of physical activity (reducing deconditioning, healthy ageing, physical and psychological health). - Fear avoidance. - JOINT SUPPORT Workbook—highlighting material on physical activity and fear avoidance.  Session 4  - Medication use and management. - JOINT SUPPORT Workbook—highlighting material on medication use and management.  Session 5  - Working through case-study scenarios to re-enforce knowledge and critique competencies on JOINT SUPPORT skills. - JOINT SUPPORT Workbook—highlighting material on case-scenarios and long-term goal setting. - Confirmation of dates for JOINT SUPPORT telephone booster calls.   The JOINT SUPPORT healthcare professional will monitor the patient–caregiver competencies, providing continual feedback and critique to support the training.  Following the 5 sessions, telephone booster calls were provided to carers and patients as a dyad at Week 1, 3 and 6 weeks after session discharged. Each telephone call took up to 20 minutes. The JOINT SUPPORT healthcare professional covered topics including:   - Recovery progress and status based on patient–caregiver shared goals. - Discussion on JOINT SUPPORT Workbook use and progress including goal-setting sheets. - Support to create collaborative goals and positive reinforcement for continued recovery. |
| **Who Provided** | A physiotherapist or occupational therapist trained in the JOINT SUPPORT programme. |
| **How** | Five, 1-hour, group-based sessions either face-to-face (3 sites) or online via video-conference calls (1 site), followed by three telephone calls at Week 1-, 3- and 6-weeks post discharge from the sessions. |
| **Where** | The programme was delivered to Physiotherapy or Occupational Therapy Departments with participants there in person or at home. |
| **When and How Much** | Five, 1-hour weekly sessions followed by 3, 20-minute booster telephone calls. |
| **Tailoring** | JOINT SUPPORT healthcare professionals were encouraged to follow the JOINT SUPPORT programme structure but to base discussions and examples of skill to develop on carer-patient participants goals, clinical and social scenarios and to personalise to the individuals in the group i.e. goal setting using an example goal of one of the patient-carer dyads. |
| **Modifications** | No modifications were made during the intervention delivery. |
| **How well: planned** | Please refer to main paper Results section. |
| **How well: actual** | Please refer to main paper Results section |

**Supplementary File 2**: Outcome measures for patient- and carer-participants

| **Patient-Participants** | **Carer-Participants** |
| --- | --- |
| - MSK-HQ [28] - Numerical rating scale (NRS) for pain [SF1] - General Self-Efficacy Scale (GSE)[SF2] - Centre for Epidemiologic Studies Depression Scale (CES-D) [SF3] - EQ-5D-5L [SF4] - Self-reported health resource use questionnaire - Adverse events | - EQ-5D-5L [SF4] - Centre for Epidemiologic Studies Depression Scale (CES-D) [SF3] - Zarit Burden Questionnaire [SF5] - Leisure Time Satisfaction questionnaire (LTS) [SF6] - Self-reported health resource use questionnaire |

**REFERENCES:**

SF1. Farrar JT, Young JP Jr, LaMoreaux L, Werth JL, Poole MR. Clinical importance of changes in chronic pain intensity measured on an 11-point numerical pain rating scale. *Pain* 2001;**94**:149-58.

SF2. Schwarzer R, J M, Weinman J, et al. Measures in health psychology: A user’s portfolio. In: Causal and control beliefs. Windsor, UK: NFER NELSON, 1995: 35–7

SF3. Radloff LS. The CES-D scale: A self-report depression scale for research in the general population. *Appl Psycholog Measurement* 1977;1:385–401.

SF4. EuroQol: EQ-5D. Available: http://www.euroqol.org/ [Accessed 08 Jul 2024]

SF5. Hébert R, Bravo G, Préville M. Reliability, validity and reference values of the zarit burden interview for assessing informal caregivers of community-dwelling older persons with dementia. *Can J Aging* 2000;**19**:494–507.

SF6. Stevens AB, Coon D, Wisniewski S, Vance D, Arguelles S, Belle S, Mendelsohn A, Ory M, Haley W. Measurement of leisure time satisfaction in family caregivers. *Aging Ment Health* 2004;**8**:450-9.

**Supplementary File 3:** Services accessed by patient-participants during the 3-month follow-up period by intervention and control group

*When N is less than 3, median and IQR not given; ^a^Other 1 includes treatments: Knee steroid injection, Obstetrics and gyne, Endocrinology eConsult; ^b^Other 2 includes treatments: Urology, Accident and Emergency

**Supplementary File 4:** Summary of the frequency in which intervention group participants (N=18) received different components of the JOINT SUPPORT programme across each treatment session.

|  | *Intervention N=18* | | | | |  |
| --- | --- | --- | --- | --- | --- | --- |
| *Item^a^* | ***N (%) Session 1*** | ***N (%) Session 2*** | ***N(%) Session 3*** | ***N(%) Session 4*** | ***N(%) Session 5*** | ***At least one occurrence (‘yes’) during sessions 1-5^b^*** |
| Provision of JOINT SUPPORT workbook  Missing | 16(100)  2 | 1 (5.9)  1 | 0  5 | 0  4 | 0  4 | 17 (100)  1 |
| Explaining pain  Missing | 16(100)  2 | 0  1 | 0  5 | 0  4 | 0  4 | 16 (100)  2 |
| Explaining caregiving  Missing | 16(100)  2 | 1 (5.9)  1 | 0  5 | 0  4 | 0  4 | 16 (100)  2 |
| How pain affects caregiver relationships  Missing | 16(100)  2 | 1 (5.9)  1 | 0  5 | 0  4 | 0  4 | 16 (100)  2 |
| Pacing  Missing | 0  2 | 17 (100)  1 | 0  5 | 1 (7.1)  4 | 0  4 | 17 (100)  1 |
| Graded activity  Missing | 0  2 | 17 (100)  1 | 1 (7.7)  5 | 1 (7.1)  4 | 0  4 | 17 (100)  1 |
| Goal setting  Missing | 0  2 | 17 (100)  1 | 1 (7.7)  5 | 0  4 | 0  4 | 17 (100)  1 |
| Physical activity  Missing | 0  2 | 0  1 | 13 (100)  5 | 0  4 | 0  4 | 13 (100)  5 |
| Fear avoidance  Missing | 0  2 | 0  1 | 13 (100)  5 | 0  4 | 0  4 | 13 (100)  5 |
| Medication use  Missing | 0  2 | 0  1 | 0  5 | 14 (100)  4 | 1 (7.1)  4 | 15 (100)  3 |
| Medication adverse effects  Missing | 0  2 | 0  1 | 0  5 | 14 (100)  4 | 1 (7.1)  4 | 15 (100)  3 |
| Case study 1  Missing | 0  2 | 0  1 | 0  5 | 0  4 | 14 (100)  4 | 14 (100)  4 |
| Case study 2  Missing | 0  2 | 0  1 | 0  5 | 0  4 | 14 (100)  4 | 14 (100)  4 |
| Case study 3  Missing | 0  2 | 0  1 | 0  5 | 0  4 | 11 (78.6)  4 | 11 (78.6)  4 |
| Confirmation of JOINT SUPPORT telephone calls  Missing | 0  2 | 0  1 | 0  5 | 0  4 | 13 (92.9)  4 | 13 (92.9)  4 |
| Other  Missing | 0  2 | 0  1 | 0  5 | 0  4 | 0  4 | 0  11 |

^a^Missing is those participants that did not attend the session. For the ‘at least one occurrence (yes)’ column, missing are those missing from all five sessions.

^b^Some patients completed the same items during multiple sessions.

**Supplementary File 5**: Dyad member session attendance summary statistics (intervention group only N=24)

| **Attending** | **At least one dyad member** | **Both dyad members** |
| --- | --- | --- |
|  | **N (%)** | **N (%)** |
| Session 1 | 18 (75.0%) | 16 (66.7%) |
| Session 2 | 19 (79.2%) | 16 (66.7%) |
| Session 3 | 15 (62.5%) | 15 (62.5%) |
| Session 4 | 15 (62.5%) | 14 (58.3%) |
| Session 5 | 17 (70.8%) | 17 (70.8%) |
| Telephone call 1  Telephone call 2  Telephone call 3 | 17 (70.8%)  18 (75.0%)  19 (79.2%) | 15 (62.5%)  17 (70.8%)  19 (79.2%) |

**Supplementary File 6**: Data completion for patient- and carer-participant at baseline and 3-month follow-up

| Outcome | Baseline Completeness  (N; %) | | 3 Month Completeness  (N; %) | |
| --- | --- | --- | --- | --- |
| Patient-Participant | **Control**  **(N=14)** | **Intervention**  **(N=24)** | **Control**  **(N=9)** | **Intervention**  **(N=18)** |
| EQ-5D-5L Index | 13 (92.9) | 24 (100) | 8 (88.9) | 18 (100) |
| EQ-5D-5L VAS | 14 (100) | 24 (100) | 8 (88.9) | 18 (100) |
| MSK-HQ total score | 14 (100) | 24 (100) | 8 (88.9) | 18 (100) |
| NRS pain | 14 (100) | 24 (100) | 8 (88.9) | 18 (100) |
| NRS fatigue | 14 (100) | 24 (100) | 8 (88.9) | 18 (100) |
| GSE total score | 13 (92.9) | 23 (95.8) | 7 (77.8) | 17 (94.4) |
| CES-D total score | 13 (92.9) | 23 (95.8) | 8 (88.9) | 18 (100) |
| Health utilisation* | 14 (100) | 24 (100) | 8 (88.9) | 18 (100) |
| Carer-Participant | **Control**  **(N=14)** | **Intervention**  **(N=24)** | **Control**  **(N=10)** | **Intervention**  **(N=18)** |
| EQ-5D-5L Index | 14 (100) | 24 (100) | 10 (100) | 18 (100) |
| EQ-5D-5L VAS | 14 (100) | 24 (100) | 10 (100) | 18 (100) |
| CES-D total score | 13 (92.9) | 23 (95.8) | 10 (100) | 18 (100) |
| Zarit Burden Interview | 13 (92.9) | 24 (100) | 10 (100) | 18 (100) |
| Leisure Time Satisfaction | 14 (100) | 21 (87.5) | 10 (100) | 17 (94.4) |
| Health utilisation* | 13 (92.9) | 22 (92.7) | 10 (100) | 18 (100) |

CES-D - Centre for Epidemiologic Studies Depression Scale; GSE - General Self-Efficacy Scale; MSK-HQ – Musculoskeletal Health Questionnaire; N – number of participants; NRS – numerical rating scale; VAS – visual analogue scale

*Completion of main seven questions (excluding subsection questions)

**Supplementary File 7:** Patient- and carer-participant outcome baseline and within group differences (3-month score minus score at individual level)

| Outcome^a^ | Outcome MCID | Baseline Data  (N; %) | | Within-Group Difference 3 Months  (N; %) | | |
| --- | --- | --- | --- | --- | --- | --- |
| Patient-Participant |  | **Control**  **(N=14)** | **Intervention**  **(N=24)** | **Control**  **(N=9)** | **Intervention**  **(N=18)** | **Favouring Group** |
| EQ-5D-5L Index | 0.08 [SF7] | 0.32 (0.27) | 0.17 (0.28) | 0.13 (0.28) | 0.06 (0.18) | Control |
| EQ-5D-5L VAS | 6.6 [SF7] | 41.6 (19.9) | 34.9 (17.8) | 7.5 (10.3) | 5.4 (24.9) | Control |
| MSK-HQ total score | 5.5 [SF8] | 48.1 (8.5) | 53.1 (8.5) | -2.4 (5.1) | -2.5 (6.0) | Similar |
| NRS pain | 1.5 [SF9] | 75 (60, 84) | 80 (70, 85) | 1.5 (-2.5, 4.0) | 0 (-5, 5) | Intervention |
| NRS fatigue | 1.4 [SF10] | 58.2 (25.4) | 76.0 (15.8) | 4.0 (13.8) | -7.4 (17.3) | Intervention |
| GSE total score | 3.0 [SF11] | 27.3 (6.3) | 25.1 (8.2) | 3.7 (3.0) | 2.4 (3.4) | Control |
| CES-D total score | 11.0 [SF12] | 23.4 (7.3) | 28.1 (10.3) | -0.6 (4.5) | -2.9 (6.0) | Intervention |
| Carer-Participant |  | **Control**  **(N=14)** | **Intervention**  **(N=24)** | **Control**  **(N=10)** | **Intervention**  **(N=18)** |  |
| EQ-5D-5L Index | 0.08 [SF7] | 0.69 (0.64, 0.77) | 0.81 (0.67, 1.00) | -0.04 (-0.18, 0.00) | 0.00 (-0.07, 0.08) | Similar |
| EQ-5D-5L VAS | 6.6 [SF7] | 63.6 (20.3) | 75.1 (20.7) | -14.6 (25.1) | 0.3 (10.4) | Intervention |
| CES-D total score | 11.0 [SF12] | 18.5 (9.0) | 18.2 (9.6) | -0.8 (8.1) | -0.5 (7.6) | Control |
| Zarit Burden Interview | Not reported | 25.9 (8.7) | 23.2 (10.8) | 1.0 (4.0) | -2.3 (6.3) | Intervention |
| Leisure Time Satisfaction | Not reported | 5.3 (3.0) | 8.4 (3.2) | 0.3 (3.4) | 0.0 (2.7) | Control |

CES-D - Centre for Epidemiologic Studies Depression Scale; GSE - General Self-Efficacy Scale; MCID – Minimal Clinically Important Difference; MSK-HQ – Musculoskeletal Health Questionnaire; N – number of participants; NRS – numerical rating scale; VAS – visual analogue scale

^a^Mean (SD) given if distribution of difference is reasonably normal in each group, otherwise median (IQR) given

**REFERENCES:**

SF7. Cheng LJ, Chen LA, Cheng JY, Herdman M, Luo N. Systematic review reveals that EQ-5D minimally important differences vary with treatment type and may decrease with increasing baseline score. *J Clin Epidemiol* 2024;**174**:111487.

SF8. Scott DIC, McCray DG, Lancaster PG, Foster PNE, Hill DJC. Validation of the Musculoskeletal Health Questionnaire (MSK-HQ) in primary care patients with musculoskeletal pain. *Semin Arthritis Rheum* 2020;50:813-20.

SF9. Young IA PT, DSc, Dunning J PT, DPT, Butts R PT, PhD, Mourad F PT, DPT, Cleland JA PT, PhD. Reliability, construct validity, and responsiveness of the neck disability index and numeric pain rating scale in patients with mechanical neck pain without upper extremity symptoms. *Physiother Theory Pract* 2019;**35**:1328-35.

SF10. Pettersson S, Lundberg IE, Liang MH, Pouchot J, Henriksson EW. Determination of the minimal clinically important difference for seven measures of fatigue in Swedish patients with systemic lupus erythematosus. *Scand J Rheumatol* 2015;**44**:206-10.

SF11. Fakontis C, Iakovidis P, Kasimis K, Lytras D, Koutras G, Fetlis A, Algiounidis I. Efficacy of resistance training with elastic bands compared to proprioceptive training on balance and self-report measures in patients with chronic ankle instability: A systematic review and meta-analysis. *Phys Ther Sport* 2023;**64**:74-84.

SF12. Haase I, Winkeler M, Imgart H. Ascertaining minimal clinically meaningful changes in symptoms of depression rated by the 15-item Centre for Epidemiologic Studies Depression Scale. *J Eval Clin Pract* 2022;**28**:500-6.

**Supplementary File 8:** Stack bar chart illustrating the patient-participant acceptability questionnaire results at 3-month follow-up

*Question Numbers:*

1. How acceptable were the JOINT SUPPORT group training sessions?
2. How acceptable were the 3 JOINT SUPPORT telephone calls?
3. How acceptable was the JOINT SUPPORT Workbook?
4. How much effort was it to engage with the JOINT SUPPORT group training sessions?
5. How much effort did it take to engage with the 3 JOINT SUPPORT telephone calls?
6. How much effort did it take to engage with the JOINT SUPPORT Workbook?
7. To what extent does the JOINT SUPPORT programme fit with your belief about managing chronic pain?
8. Is the JOINT SUPPORT programme likely to change your ability to manage your chronic pain?
9. Does the JOINT SUPPORT programme provide you with more confidence on managing your chronic pain?
10. Is it clear how the JOINT SUPPORT programme could help you manage your chronic pain?
11. Did doing the JOINT SUPPORT programme interrupt with your other priorities?
12. Did doing the JOINT SUPPORT study interrupt with your other priorities?

*Score equate to:*

Question 1-3: (1) completely unacceptable; (2) unacceptable; (3) neither acceptable nor unacceptable; (4) acceptable; (5) completely acceptable

Question 4-6 :(1) huge effort; (2) a lot of effort; (3) none of these; (4) a little effort; (5) no effort at all

Question 7: (1) strongly does not fit with belief; (2) does not fit with belief; (3) neither fits or unfits with belief; (4) fits with belief; (5) strongly fits with belief.

Question 8: (1) Very unlikely to change; (2) unlikely to change; (3) neither likely or unlikely to change; (4) likely to change; (5) very likely to change.

Question 9: (1) very unconfident; (2) unconfident’ (3) neither confident or unconfident’ (4) confident’ (5) very confident.

Question 10: (1) very unclear; (2) unclear; (3) neither clear or unclear; (4) clear; (5) very clear

Question 11-12: (1) strongly agree; (2) agree; (3) neither agree or disagree; (4) disagree; (5) strongly disagree

**Supplementary File 9:** Stack bar chart illustrating the carer-participant acceptability questionnaire results at 3-month follow-up

*Question Numbers:*

1. How acceptable were the JOINT SUPPORT group training sessions?
2. How acceptable were the 3 JOINT SUPPORT telephone calls?
3. How acceptable was the JOINT SUPPORT Workbook?
4. How much effort was it to engage with the JOINT SUPPORT group training sessions?
5. How much effort did it take to engage with the 3 JOINT SUPPORT telephone calls?
6. How much effort did it take to engage with the JOINT SUPPORT Workbook?
7. To what extent does the JOINT SUPPORT programme fit with your belief about managing chronic pain?
8. Is the JOINT SUPPORT programme likely to change your ability to manage your chronic pain?
9. Does the JOINT SUPPORT programme provide you with more confidence on managing your chronic pain?
10. Is it clear how the JOINT SUPPORT programme could help you manage your chronic pain?
11. Did doing the JOINT SUPPORT programme interrupt with your other priorities?
12. Did doing the JOINT SUPPORT study interrupt with your other priorities?

*Score equate to:*

Question 1-3: (1) completely unacceptable; (2) unacceptable; (3) neither acceptable nor unacceptable; (4) acceptable; (5) completely acceptable

Question 4-6 :(1) huge effort; (2) a lot of effort; (3) none of these; (4) a little effort; (5) no effort at all

Question 7: (1) strongly does not fit with belief; (2) does not fit with belief; (3) neither fits or unfits with belief; (4) fits with belief; (5) strongly fits with belief.

Question 8: (1) Very unlikely to change; (2) unlikely to change; (3) neither likely or unlikely to change; (4) likely to change; (5) very likely to change.

Question 9: (1) very unconfident; (2) unconfident’ (3) neither confident or unconfident’ (4) confident’ (5) very confident.

Question 10: (1) very unclear; (2) unclear; (3) neither clear or unclear; (4) clear; (5) very clear

Question 11-12: (1) strongly agree; (2) agree; (3) neither agree or disagree; (4) disagree; (5) strongly disagree

**Supplementary File 10:** Barriers, facilitators and suggested improvements to the JOINT SUPPORT study, as suggested by participants (patients, carers and health professionals)

| **Patient-Carer Participants** | **Health Professional-Participants** |
| --- | --- |
| **Barriers** | |
| - Time commitment required to attend all sessions, - Capacity to be involved (‘to be present in body & mind’), - Dyad dynamics (nature of relationship), - Hospital setting (including travel burden), - Technical issues when joining online sessions - Timing of sessions (to accommodate for symptoms of pain, such as fatigue). | - Nuances and complexities of the NHS system (fragmented nature of the pain services), - Too broad of an inclusion criteria, - Accommodating the needs of patients (more than just pain) - Administrative activities (managing clinical priorities) - DNA phone calls (‘time wasting’), - Group dynamics (time to build trust and rapport) - Dyad dynamics (variability in relationships, dominant/conditioned speaker among the dyad), - Variability in conditions. |
| **Facilitators** | |
| - High level of rapport between dyads and health professionals (built on the health professional’s personable characteristics and their knowledge of pain management), - Group-based sessions (opportunity for socialisation), - Group cohesion (sharing experiences was validating, comforting and encouraging for dyads). | - Readiness to deliver the intervention (thorough training and support), - A physical presence in clinics/waiting rooms supported recruitment, - Tailored communication to suit dyads, - Group cohesion (facilitating rapport among the group), - Practical/’real-life’ activities and discussions (i.e., case studies). |
| **Suggestions for Improvement** | |
| - Online platforms to house study information and provide consent, - Options for online or hybrid participation in group sessions, - Separate workbooks and sessions designed and tailored to both parts of the dyad, - Use of Apps for data collection, - Condition-specific group sessions. | - Engaging more staff in the recruitment process (raising awareness beyond the immediate research team), - Online platforms to house study information and provide consent, - People with lived experience of pain to deliver health professional training (to promote the authenticity of scenarios), - Access to recorded material to top-up/refresh training, - Options for online or hybrid participation in group sessions, - Inclusion of ‘stress management’ as a topic to cover in sessions and the workbook, - Options for ‘out-of-hour’ sessions to support carer involvement (e.g., those in employment), - Online, supplementary material for dyads to access post-session (to consolidate learning). |
