# Supplementary material for: Pain management training for people with persistent pain and their informal carers (JOINT SUPPORT): multicentre randomised controlled feasibility trial with embedded qualitative study in English musculoskeletal services
Source: BMJ Open. 2025 Apr 15;15(4):e095069. doi: 10.1136/bmjopen-2024-095069 (PMC12001371; doi:10.1136/bmjopen-2024-095069)
Supplement: Supplementary file 2 [file bmjopen-2024-095069.reviewer_comments.pdf]

PEER REVIEW HISTORY

BMJ Open publishes all reviews undertaken for accepted manuscripts. Reviewers are asked to complete a checklist review form and are provided with free text boxes to elaborate on their assessment. These free text comments are reproduced below.

ARTICLE DETAILS

Title (Provisional)

Pain management training for people with persistent pain and their informal carers (JOINT SUPPORT): multicentre randomised controlled feasibility trial with embedded qualitative study in English musculoskeletal services.

Authors

Smith, Toby; Khoury, Reema; Welsh, Allie; Crowther, Coralie; Hanson, Sarah; Grant, Kelly; Clark, Allan B; Ashford, Polly-Anna; Hammond, Matthew; Pond, Martin; Dures, Emma; Adams, Jo

---

VERSION 1 - REVIEW

Reviewer 1

Name Allam , Nesma M.

Affiliation Department of physical therapy for surgery, Faculty of Physical Therapy, Cairo University

Date 04-Nov-2024

COI None

I appreciate your great efforts for this great work. I found the paper to be overall well written and much of it to be well described. But I have some comments:

1-Abstract:

- Add some details about the results.

2- Introduction:

- Write ESCAPE abbreviation in full words as it is the first time to be mentioned in the text.

- What is the rationale for your study?

3-Methods:

- In inclusion criteria, determine the cause and the level of pain.

- It is suggested to add more details about JOINT SUPPORT program in a supplementary material.

- In outcome measures, it is better to be written in a paragraph instead of points.

- Add the power value, and effect size for the sample size.

4- Results:

- What are the statistical tests used in this study?

5-Discussion:

- Discuss your results with more previous studies to strengthen your study.

Reviewer **2**

Name **Ryan, Cormac**

Affiliation **Teesside University**

Date **18-Dec-2024**

COI **I have previous collaborated with the lead author in a professional capacity and we once sat on the same committee, however this was several years ago. We do not routinely work together, and have met very rarely in the past 5 years. I do not believe that this conflict of interest presents a significant issue to my reviewing of this work.**

Thank you for the opportunity to review this interesting and important piece of work. I have provided some minor comments below which I hope the authors will find helpful.

Abstract

Page 4:

The strengths and limitations of the study. The final bullet point “Perceptions of family members…” This point could be clearer, perceptions remain unclear is quote vague for n=14 interviews, as an overall point. Consider revising.

Introduction

Page 5:

The final sentence in paragraph 2 says quite bluntly that being a carer is a burden, which is a very narrow view and potentially a little offensive to those with pain who have a carer suggestive that they are only a burden to others. I fully appreciate that this will not have been intentional by the authors. Can I recommend that this sentence be revised to highlight that caring can be burdensome but highlight that it can also be rewarding.

Methods:

Page 5:

It looks like having persistent pain was qualified as having pain for 6 weeks or more. Most definitions use three months as the cut-off. Please provide a justification within the text as to why 6 weeks was used.

Page 6:

Please add a link to a supplementary table reporting the intervention using the TiDiER framework.

Page 7:

It is stated that blinding was not possible and lists reasons. I am not sure I agree with this statement, these issues make blinding difficult but not impossible, and creative solutions could be explored going forward into the full trial. Please consider rephrasing that blinding was not undertake because of the following… Then in the discussion section add some sentences about how it could potentially be done e.g. for participant blinding and attention control could be added where the pair as a group get relevant but non-therapeutic information.

Page 8:

The final paragraph, I think Sha should be SHA or maybe just SH for consistency of how other authors have been abbreviated in the text.

Whilst the reference describing how the qualitative analysis was undertaken are helpful, some details of the process in the text would be helpful to the reader, 3-4 lines should suffice.

Results:

Page 9:

The authors state "Six participants did not receive the intervention, therefore 18 participant data were reported." This statement seems odd to me – do you mean therefore data for 18 participant collection points were not reported? Please clarify.

Only one participant completed the carer log – this issue could be discussed a little more in the main text of the discussion with regards to what that tells us about adherence and fidelity. It gives the impression that the carers were not very engaged. If this is the case it implies that potentially what we are seeing here is an intervention being provided to a patient who happens to bring along another individual who is just a plus 1 rather than an engaged individual. A little more discussion of this I think is warranted.

Discussion:

Page 13 paragraph 3 – it is noted that one hospital offered online sessions and this may help/have helped with recruitment. Given the importance of recruitment and indeed adherence and engagement, it would be useful to explore this issue a little more in this paragraph and quantify some key metrics such as what was the difference in uptake

between the hospital that offered online and the other hospitals. And at the hospital that offered online, what was the break down in the choice between in person and online.

Supplementary file 3 – this table was a little confusing for me as it was unclear whether the different interventions should have occurred in each session or not e.g. for education 16 (100%) got it in session 1 but it does not look like it was covered after that. Was that because it was only meant to be discussed in session 1 or was it missed by mistake in later sessions. I think this table needs a little more explanation, maybe a more detailed legend?

Supplementary file 6 – interpretation of this table would benefit from adding an MCID for each of the measures.

VERSION 1 - AUTHOR RESPONSE

Reviewer 1

Comment: I appreciate your great efforts for this great work. I found the paper to be overall well written and much of it to be well described. But I have some comments:

Response: Thank you for your helpful comments. We have addressed the comments below.

Comment: Abstract: Add some details about the results.

Response: As requested, we have now provided patient-participant clinical outcome results into the abstract. However, due to the underpowered nature of these findings (as this was a feasibility study), we have presented this solely as a descriptive illustration of clinical findings (**Abstract, Results, Lines 4-7**).

Comment: Introduction: Write ESCAPE abbreviation in full words as it is the first time to be mentioned in the text.

Response: This has now been added (**Introduction, Paragraph 3, Line 3-4**).

Comment: Introduction: What is the rationale for your study?

Response: As requested, we have now provided a more robust and explicit rationale for the study (**Introduction, Paragraph 3, Line 7-11**).

Comment: Methods: In inclusion criteria, determine the cause and the level of pain.

Response: Our study did not specify the cause or level of pain in the eligibility criteria as outlined in the published protocol and trial registration (Smith et al, 2020). We are therefore unable to amend what was adopted in practice. However, to improve clarification on eligibility, we have provided a more detailed report on the eligibility criteria in the study (**Methods, Eligibility Criteria, Bullet Points 1-6**).

Comment: Methods: It is suggested to add more details about JOINT SUPPORT program in a supplementary material.

Response: As requested by Reviewer 1 and Reviewer 2, we have provided further details on the JOINT SUPPORT programme, reported in accordance with the TIDieR reporting checklist

(**Supplementary File 1**). This is cross-reference to the main text (**Methods, Study Treatments, Paragraph 2, Lines 1-2**).

Comment: Methods: In outcome measures, it is better to be written in a paragraph instead of points.

Response: As requested, we have presented this text as a paragraph rather than bullet points (**Methods, Outcome Measures, Lines 1-21**).

Comment: Add the power value, and effect size for the sample size.

Response: As this is a feasibility study, the trial design does not require a formal power calculation to determine power for a specific clinical outcome. Therefore, no specific power calculation has been presented. However, we have provided more justification for the sample size in the revised paper to offer the reviewer more justification for our approach (**Methods, Sample Size, Line 3-6**).

Comment: Results: What are the statistical tests used in this study?

Response: As this was a feasibility study, no statistical tests were planned nor conducted for the study, given the design is not appropriate to test for statistical difference. This has been stated in the revised paper (**Methods, Data Analysis and Progression Criteria, Paragraph 1, Line 5-6**).

Comment: Discussion: Discuss your results with more previous studies to strengthen your study.

Response: There has been limited evidence published on carer-patient training or dyadic interventions within the literature. Nonetheless, we have incorporated a paragraph into the Discussion, as recommended by Reviewer 1, to both present and compare the clinical results of this study, with the one available previous published paper of a comparable intervention and have also highlighted the paucity of evidence in the field (**Discussion, Paragraph 5, Lines 1-9**).

<u>**Reviewer 2**</u>

Comment: Thank you for the opportunity to review this interesting and important piece of work. I have provided some minor comments below which I hope the authors will find helpful.

Response: Thank you for your helpful comments. We have addressed the comments below.

Comment: Abstract: Page 4: The strengths and limitations of the study. The final bullet point "Perceptions of family members…" This point could be clearer, perceptions remain unclear is quote vague for n=14 interviews, as an overall point. Consider revising.

Response: We have clarified this point as a weakness with the revision that the unexplained nature of variation in perspectives remains a limitation which this study was unable to answer. This has been revised in the paper (**Strengths and limitations of the study, Bullet Point 5**).

Comment: Introduction: Page 5: The final sentence in paragraph 2 says quite bluntly that being a carer is a burden, which is a very narrow view and potentially a little offensive to those with pain who have a carer suggestive that they are only a burden to others. I fully appreciate that this

will not have been intentional by the authors. Can I recommend that this sentence be revised to highlight that caring can be burdensome but highlight that it can also be rewarding.

Response: Thank you for highlighting this very important point. We agree with the reviewer's positioning and have made an amendment to reflect this agreement in the revised paper (**Introduction, Paragraph 2, Lines 12-15**).

Comment: Methods: Page 5: It looks like having persistent pain was qualified as having pain for 6 weeks or more. Most definitions use three months as the cut-off. Please provide a justification within the text as to why 6 weeks was used.

Response: The reviewer is correct in flagging this. A six-week cut point was used as the trajectory of establishment of persistent pain is suggestive of occurring at 6 weeks and this also corresponds to guidance on when people should begin to seek treatment for pain which has not resolved. This has been reflected in the revised eligibility criteria (**Methods, Inclusions Criteria, Bullet Point 1**).

Comment: Page 6: Please add a link to a supplementary table reporting the intervention using the TiDiER framework.

Response: As requested by Reviewer 1 and Reviewer 2, we have provided further details on the JOINT SUPPORT programme, reported in accordance with the TIDieR reporting checklist (**Supplementary File 1**). This is cross-reference to the main text (**Methods, Study Treatments, Paragraph 2, Lines 1-2**).

Comment: Page 7: It is stated that blinding was not possible and lists reasons. I am not sure I agree with this statement, these issues make blinding difficult but not impossible, and creative solutions could be explored going forward into the full trial. Please consider rephrasing that blinding was not undertake because of the following... Then in the discussion section add some sentences about how it could potentially be done e.g. for participant blinding and attention control could be added where the pair as a group get relevant but non-therapeutic information.

Response: We have revised both the Methods section to amend impossible to challenging, as recommended (**Methods, Randomisation and Blinding, Paragraph 2, Line 1**), and also flagged this within the Discussion and a point for consideration in future trial designs (**Discussion, Paragraph 6, Line 14-21**).

Comment: Page 8: The final paragraph, I think Sha should be SHA or maybe just SH for consistency of how other authors have been abbreviated in the text.

Response: We have corrected this as recommended (**Methods, Embedded Qualitative Study, Paragraph 5, Line 3**).

Comment: Whilst the reference describing how the qualitative analysis was undertaken are helpful, some details of the process in the text would be helpful to the reader, 3-4 lines should suffice.

Response: As requested, we have provided further details on the qualitative analysis methods (**Methods, Embedded Qualitative Study, Paragraph 4, Line 1-9**).

Comment: Results: Page 9: The authors state "Six participants did not receive the intervention, therefore 18 participant data were reported." This statement seems odd to me – do you mean therefore data for 18 participant collection points were not reported? Please clarify.

Response: We have clarified that 18 of the 24 participant-dyads allocated to the JOINT SUPPORT programme received the intervention (**Results, Intervention Fidelity (Health Professionals), Paragraph 1, Lines 1-2**).

Comment: Only one participant completed the carer log – this issue could be discussed a little more in the main text of the discussion with regards to what that tells us about adherence and fidelity. It gives the impression that the carers were not very engaged. If this is the case it implies that potentially what we are seeing here is an intervention being provided to a patient who happens to bring along another individual who is just a plus 1 rather than an engaged individual. A little more discussion of this I think is warranted.

Response: Thank you for highlighting this. We agree that this provides another indication on the challenges of carer-perception either to their role, the intervention or study procedures. We have outlined in Paragraph 5 (Discussion) the potential issues around carer-perception to the trial more widely and incorporated carer log as another example as suggested (**Discussion, Paragraph 6, Line 6-7**). We have not been able to elaborate further, being mindful of the paper's word count restriction but feel this sentiment is communicated, as highlighted by the reviewer, in this paragraph.

Comment: Discussion: Page 13 paragraph 3 – it is noted that one hospital offered online sessions and this may help/have helped with recruitment. Given the importance of recruitment and indeed adherence and engagement, it would be useful to explore this issue a little more in this paragraph and quantify some key metrics such as what was the difference in uptake between the hospital that offered online and the other hospitals. And at the hospital that offered online, what was the break down in the choice between in person and online.

Response: Thank you for highlighting this increasingly important point. There were no key differences in recruitment rate, retention numbers nor characteristics of the cohort from Site 4, compared to Sties 1-3. However, as the numbers were small, it was felt inappropriate to present subgroup analyses by site for these metrics. Nonetheless, we have provided this overview in the Discussion, so the reader is made aware of this important point (**Discussion, Paragraph 4, Line 5-8**).

Comment: Supplementary file 3 – this table was a little confusing for me as it was unclear whether the different interventions should have occurred in each session or not e.g. for education 16 (100%) got it in session 1 but it does not look like it was covered after that. Was that because it was only meant to be discussed in session 1 or was it missed by mistake in later sessions. I think this table needs a little more explanation, maybe a more detailed legend?

Response: Apologies for the confusion. The purpose of this table was to illustrate what components of the programme were delivered and when. It was anticipated that people would receive the components in sequential order i.e. early components delivered in sessions 1 and 2 with later components delivered in sessions 3 to 5. To address this, we have provided further information in the paper through **Supplementary File 1** TIDieR checklist and have revised the legend as suggested by the reviewer (**Figure and Table Legends, Supplementary File 4 – Legend**).

Comment: Supplementary file 6 – interpretation of this table would benefit from adding an MCID for each of the measures.

Response: As requested, this is provided for all outcomes in Supplementary File 6 with the exception of Leisure Time Satisfaction and Zarit Burden Interview scale which has not been reported in the literature for this population (**Supplementary File 7, Outcome MCID – Column 2**).
